# Supplementary material for: Enhancing CRISPR-Cas9 gRNA efficiency prediction by data integration and deep learning
Source: Nat Commun. 2021 May 28;12:3238. doi: 10.1038/s41467-021-23576-0 (PMC8163799; doi:10.1038/s41467-021-23576-0)
Supplement: Supplementary file 1 — Supplementary Information [file 41467_2021_23576_MOESM1_ESM.pdf]

Supplementary information

Enhancing CRISPR-Cas9 gRNA efficiency prediction by data integration and deep learning

Xi Xiang[1-4*], Giulia I. Corsi[5*], Christian Anthon[5*], Kunli Qu[1,11*], Xiaoguang Pan[1], Xue Liang[1,11], Peng Han[1,11], Zhanying Dong[1], Lijun Liu[1], Jiayan Zhong[6], Tao Ma[6], Jinbao Wang[6], Xiuqing Zhang[3], Hui Jiang[6], Fengping Xu[1,3], Xin Liu[3], Xun Xu[3,7], Jian Wang[3], Huanming Yang[3,8], Lars Bolund[1,3,4], George M. Church[9], Lin Lin[1,4,10], Jan Gorodkin[5,‡] & Yonglun Luo[1,3,4,10,‡]

[1]Lars Bolund Institute of Regenerative Medicine, Qingdao-Europe Advanced Institute for Life Sciences, BGI-Qingdao, Qingdao 266555, China.

[2]BGI Education Center, University of Chinese Academy of Sciences, Shenzhen 518083, China.

[3]BGI-Shenzhen, Shenzhen 518083, China.

[4]Department of Biomedicine, Aarhus University, Aarhus 8000, Denmark.

[5]Center for non-coding RNA in Technology and Health, Department of Veterinary and Animal Sciences, Faculty of Health and Medical Sciences, University of Copenhagen, Frederiksberg 1871, Denmark.

[6]MGI, BGI-Shenzhen, Shenzhen 518083, China.

[7]Guangdong Provincial Key Laboratory of Genome Read and Write, BGI-Shenzhen, Shenzhen, 518120，China

[8]Guangdong Provincial Academician Workstation of BGI Synthetic Genomics, BGI-Shenzhen, Shenzhen, 518120，China

[9]Department of Genetics, Blavatnik Institute, Harvard Medical School, Boston, MA 02115, USA

[10]Steno Diabetes Center Aarhus, Aarhus University, Aarhus 8200, Denmark

[11]Department of Biology, University of Copenhagen, Copenhagen N 2200, Denmark.

*These authors contributed equally: Xi Xiang, Giulia I. Corsi, Christian Anthon, Kunli Qu

‡These authors jointly supervised this work: Jan Gorodkin (computational), Yonglun Luo (experimental). Email: gorodkin@rth.dk, alun@biomed.au.dk

Supplementary Note 1: The advance in CRISPR gRNA activity prediction is mostly data-driven, rather than model-driven

Numerous machine learning methods have been so far applied for predicting the editing efficiency of SpCas9 gRNAs, including linear regressors[1-3], SVMs[4-9], tree-based models[8, 10] and deep-learning networks[11-14]. Among notable predictors, the gradient boosting regressor "Azimuth"[10] has for long been known because of its superior generalization ability in predicting SpCas9 efficiency for gRNAs expressed from a U6 promoter, as highlighted by the independent benchmark of Haeussler *et at.*[15]. A further advance was recently offered by the deep learning regressors of Kim *et al.*, "DeepSpCas9", and Wang *et al.*, "DeepHF", trained on large-scale datasets of SpCas9 efficiencies generated by the same groups[12, 13]. Even more recently, Kim *et al*. developed a new set of deep-learning regression models, "DeepSpCas9variants", which predicts gRNA efficiency in relation with SpCas9 and eight variants of the complex with remarkable prediction ability[14]. Below, we show that the performance of this model was overestimated by mixing together efficiency data derived from both canonical and non-canonical PAMs, with the latter being easy to predict because of their general low efficiency. Moreover, we explain that the improvement of a deep learning model such as DeepSpCas9 over a regular machine learning tool such as Azimuth, is to be attributed predominantly to the usage of a larger training dataset and not to the application of a more complex model.

According to the comparison among machine learning-based methods presented by Kim *et al.*[12], tree-based regressors, including a gradient boosting regression tree (GBRT) resembling Azimuth, were the worst performing models in a 10-fold cross-validation and in additional independent tests, a result profoundly in contrast with the previous analysis of Doench *et al.*[10]. We reasoned that this discrepancy might be caused by an unfortunate choice of validation hyperparameters. In the study by Kim *et al.*, tree-based models were configured to have a maximum depth of at least 50, a minimum number of samples necessary for branching of 2 or 4 and a minimum number of samples in leaf nodes of 1 or 2, while no tuning was performed on the learning rate. Such hyperparameters might generate considerably complex regression trees, which are not suitable for an ensemble model designed to combine weak learners rather than powerful predictors[16].

To address this, we repeated the training of GBRTs on the Kim *et al.* (2019) by decreasing the tree complexity directed by hyperparameters, using input features either as in Azimuth or as in DeepSpCas9 (only sequence-related). We validated 432 GBRTs on the dataset of Kim *et al.* (2019) with the following hyperparameters: learning rate chosen from [0.05, 0.08, 0.1], maximum tree depth chosen from [3, 5, 7, 10], minimum number of samples to generate a new split chosen from [10, 15, 20], minimum number of samples to be present in a leaf node chosen from [3, 5, 7], total number of trees in the model chosen from [200, 400, 600, 800]. The structure and input features of the trees was the same described in Material and Methods for CRISPRon-GBRT v0 and v1, with either the same features except ΔG_B, to resemble Azimuth, or only the sequence-related ones. In lack of information on the cross-validation set construction by Kim *et al.*, we divided the dataset in 11 subsets accounting for data similarity, such that highly similar gRNAs were grouped in the same subset (see Methods). Hyperparameter cross-validation was performed on 10 subsets, while the remaining one was preserved as an independent test set. Additionally, we validated 192 GBRTs with the same hyperparameters chosen by Kim *et al.* (max depth = 600 was employed to approximate training until leaf purity) and re-trained their deep learning-based model in a cross-validation over the 10 validation subsets. This latter model, referred to as DeepSpCas9-Val, had the same performances as the one reported for the validation of the original DeepSpCas9. The GBRTs trained with our selection of hyperparameters significantly outperformed those of Kim *et al.* and did not present any significant prediction difference compared to the re-validated DeepSpCas9 on the internal independent test set (Supplementary Fig. 1). We then compared the re-trained GBRTs and the original DeepSpCas9 model on the external independent datasets targeting human cells analyzed by Kim *et al.*[1, 4, 10, 17-19]. The datasets were pre-processed and filtered as explained in Methods (main text), except for the gRNAs targeting the last 10% of the merged coding sequence of the target gene, which were not excluded in this comparison. We report that the GBRTs and deep learning-based model performed equally well on the external independent test set (removal of similarity with training data was performed as explained in Methods, see main text). Importantly, the appropriate re-training of Azimuth enabled to achieve performance results comparable to DeepSpCas9. Hence, we ascribed the prediction improvement observed by Kim *et al.* to the large high-quality dataset generated in their study, rather than their deep learning model. In agreement with this, we also observe a modest difference between the

CRISPRon (v0 and v1) deep learning-based model and the CRISPRon-GBRT (v0 and v1) trained on the same dataset (Supplementary Data 2).

Supplementary Note 2: Evaluating gRNA predictions on data from different PAMs leads to performance overestimation

The recent DeepSpCas9variants model[14] was trained and tested on both canonical and non-canonical PAMs, and because of this we suspected its reported performances to be inflated by the presence of numerous zero-value gRNAs, easy to predict because of their PAM composition. Indeed, the PAM was also the feature reported to have the highest SHAP importance in the related study. We tested DeepSpCas9variants after removing one PAM at a time, from the lowest to the highest efficient one. We observed a consistent decrease in performances from a Spearman correlation $R=0.94$ and a Pearson's $r=0.96$ down to $R=0.70$ and $r=0.79$, with the latter measure obtained when exclusively considering the canonical NGG PAM (Supplementary Fig. 1). Moreover, DeepSpCas9variants showed scarce generalization ability compared to its predecessor DeepSpCas9 and other models when benchmarked on external independent test datasets (Supplementary Data 2), suggesting that its training data has characteristics that were not present in previous studies. These characteristics go beyond the presence of non-canonical PAMs in the training dataset, which are easily identified and categorized by machine learning, as shown by the SHAP analysis mentioned above. None of DeepSpCas9, DeepHF, pre-CRISPRon_v1 or Azimuth could predict the gRNA efficiencies reported in the dataset used to train DeepSpCas9variants (Kim *et al*. (2020), filtered for NGG PAMs only) with a Spearman's R > 0.5, and this dataset was the lowest in terms of predictions for all models except DeepSpCas9, trained on data generated by the same group, for which it was the third lowest (Supplementary Data 2). Instead, the gRNA efficiency values of both our data and Kim *et al*. (2019) were exceptionally well predicted by both DeepSpCas9 and pre-CRISPRon_v0 (Spearman's R >0.7). Thus, despite its size, the Kim *et al.* (2020) dataset was excluded for the development of CRISPRon.

Supplementary Note 3: Establishment of the lentiviral surrogate vector-based gRNA efficiency evaluation method

We and several other groups previously demonstrated that a surrogate target site can faithfully recapitulate the endogenous editing efficiency and indel profile. To streamline vector cloning, accurate quantification of viral titer, and enrichment of gene edited cells, we firstly designed a lentivirus-based system with three main features (plasmid can be acquired from the Luo lab): (1) Golden-Gate Assembly (GGA) based cloning with a lac Z marker for precise and efficient insertion of the gRNA expression cassette; (2) A green fluorescent protein (GFP) marker for measuring viral titer and real-time tracking of viral delivery; (3) A puromycin selection gene for enrichment of stably transduced cells (Supplementary Fig. 2). Essentially, this lentivirus system allows conventional GGA-based insertion of a synthetic DNA containing a gRNA spacer, scaffold and the corresponding surrogate target site after the U6 promoter. As current microarray-based method can only faithfully synthesize oligo pools of max 170 bp, we optimized the DNA design to contain a 102bp gRNA expression cassette (20bp spacer + 82bp scaffold) and a 37bp surrogate target site, flanked by 31bp GGA cloning sites and PCR handles (Fig. 1a, Supplementary Fig. 2). Although the surrogate site is 37bp, we validated at 17 surrogate and corresponding endogenous sites that there is a good correlation in on-target gRNA efficiency between them.

Supplementary Note 4: Massively parallel quantification of on-target gRNA efficiency

Several experimental procedures have to be optimized to generate the 12K sequencing library. A detail protocol is shared in protocols.io[20]. PCR conditions have to be optimized by gradient melting temperature and PCR cycles. With optimized PCR conditions, we have setup **72** parallel PCR reactions (20ul in each reaction) and the final PCR products were pooled and purified. All these detail steps are to faithfully amplify the 12,000 oligos from pooled oligos without causing PCR-induced bias. Next, for Golden-Gate Assembly, it is essential to perform large replicates to avoid the ligation-induced bias in oligo representation as well. In our optimized condition, we have performed 36 independent GGA reactions (20ul per reaction). For the same reason, 42 independent *E.coli* transformations with GGA ligation product were performed (10ul each). Our deep

sequencing results of the 12K pool oligos, 12K plasmid library and 12K lentivirus library transduced cells prove that the optimized procedure yield high coverage (over 99%) and correlation (Pearson's r = 0.86-0.91).

For on-target gRNA efficiency, we transduced the HEK293T expressing the codon-optimized SpCas9 with the 12K lentivirus (MOI is 0.3 and transduction coverage is approximately 4000 cells per surrogate site. Based on this, the number of cells used for transduction is 12K * 4000/MOI. The expression of SpCas9 was controlled by a Doxycycline (Dox)-inducible TRE promoter. However, due the leakiness of promoter activity, we can observe significant SpCas9 activity in the cells without addition of Dox. Enhanced SpCas9 expression by Dox addition significantly increase the on-target editing efficiency leading to over skewed and saturated on-target gRNA activities (Supplementary Data 1, S7). Thus, for CRISPRon model training, we only used on-target gRNA efficiency data from the HEK293T-SpCas9 cells without Dox addition.

Supplementary Note 5: The dynamics and predictable characteristics of indels introduced by SpCas9

Repair of the double-strand DNA breaks (DSBs) introduced by SpCas9 is carried out by the endogenous DSB repair machineries, and mostly by the microhomology-mediated end-joining (MMEJ) and non-homologous end-joining (NHEJ) repair pathway. While indel profiles differ between DSBs introduced by different gRNAs, it has been found that the repair outcomes (indel profiles) are predictable and approximately 5–11% SpCas9 gRNAs induced a dominant indel (>50% of all indel events)[21]. One dominant indel type is 1-bp insertion in the DSB site. Besides, it has also been showed that, depending on the CRISPR/Cas9 delivery formats (plasmids transfection, lentiviral transduction, ribonuclear protein electroporation) and time, there exists certain dynamics in indel profiles. Under conditions of persistent editing (via stable integration of SpCas9 and gRNA in cells), relative higher frequency of small indels (1-bp deletion and 1-bp insertion) appear earlier post expression of SpCas9 and gRNA. Importantly, the frequency of large indels (> 6 bp) increase following persistent editing and reach a stable distribution of indel types. Consistent with that, our data showed that 1-bp deletion and 1-bp insertion are the two most

frequent indel types across all time points studied (Fig. 1d and Supplementary Fig. 7). The frequency of other indels (> 2 bp) increase following increased editing time and over-expression of SpCas9 (Fig. 1d and Supplementary Fig. 7). The inDelphi was developed based on indel profile dataset from cells with one-week persistent editing of over-expressing SpCas9. We validate our gRNA efficiency evaluation approach by comparing the indel profiles of our gRNAs to the corresponding indel profiles predicted by inDelphi. Our results showed that correlations were increased from day 2 to day8-10 (Fig. 1e and Supplementary Fig. 7). When analyzing the correlation between nucleotides of the 1-bp insertion indel and nucleotides presented at the N17 position (4-nt upstream of the PAM), corroborating with the finding by Shen et al. our results showed that the 1-bp inserted nucleotide is mostly identical to the N17 nucleotide (Fig. 1f and Supplementary Fig. 7). Besides, the presence of T at N17 favors insertional indels, while the presence of G at N17 more favors deletion indels (Supplementary Fig. 7). Though more studies are needed to better understand the mechanism of these predictive features and indel pattern, this "partially" predictive indel profiles allow us to introduce or correct mutations without donor templates.

Supplementary Note 6: Analysis of features important for CRISPR gRNA on-target activity prediction

To characterize the features with the highest contribution to the learning process we employed two strategies: the Shapley Additive exPlanations (SHAP)[22] and the Gini importance[23] (Supplementary Fig. 12). Both methods are applied on a gradient boosting regression tree (GBRT) version of CRISPRon, CRISPRon-GBRT (v0 and v1), whose performances exceeded those of existing models similarly to CRISPRon_v0 and v1 (Supplementary Data 2). The SHAP method reflects the GBRT model onto individual training instances to explain predictions by computing the local contribution of each feature to the predicted value. The global importance of a feature is then obtained by summarizing the local contributions over the whole training dataset. In complement, the Gini importance explains the composition of a GBRT globally in terms of variance reduction achieved by splitting the data in a node based on a given feature and can thus be considered as a reflection of the training data onto the model. Among the top 20 most relevant features highlighted by both methods within their top 25, thermodynamic properties such as ΔG_B, MFE and melting

temperatures dominate. A few sequence-composition features also resulted to be highly relevant, particularly in the seed region.

Supplementary Note 7: Data integration requires a fully representative gRNA activity distribution

The large-scale dataset of SpCas9 gRNA efficiencies generated by Wang *et al.* (2019)[13] was omitted from the training of CRISPRon because the choice of selecting gRNAs based on the Azimuth's predictions resulted in a skewed distribution of gRNAs efficiencies, which is not fully representative of the CRISPR gRNAs landscape and would create a bias in our data integration process (Supplementary Fig. 1).

a

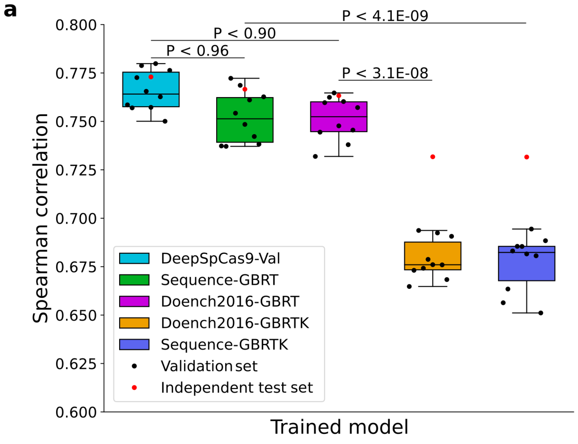

b

	DeepSpCas9	Sequence-based GBRT	Doench 2016 GBRT
Doench 2014 (*n* = 106)	0.609	0.681*	0.681[NS]
Xu 2015 (n = 1829)	0.583	0.584[NS]	0.588[NS]
Chari 2015 (*n* = 1198)	0.450	0.448[NS]	0.443[NS]
Kim-Test 2019 (*n* = 527)	0.773	0.767[NS]	0.748[NS]
Hart 2015 (*n* = 4158)	0.462	0.455[NS]	0.459[NS]
Doench 2016 (*n* = 427)	0.415	0.406[NS]	0.406[NS]

Spearman correlation 0.0 0.4 8.0

c **d**

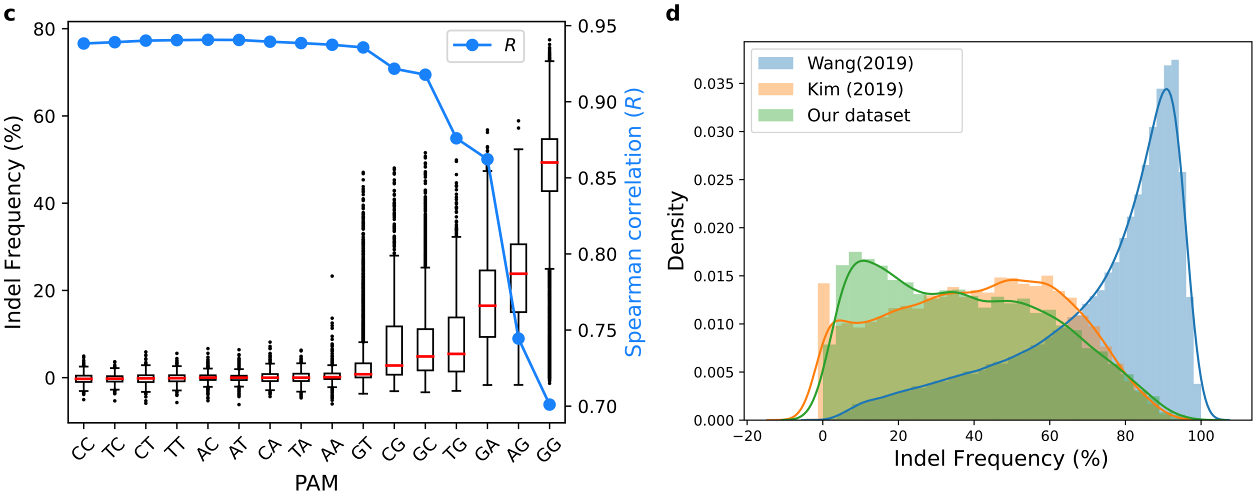

Supplementary Figure 1. Evaluation of recent datasets and models of CRISPR gRNA efficiency. a. Comparison between the validation performances of different ML models on Kim et al. (2019) dataset. Black and red dots correspond to the Spearman correlation between experimentally measured and predicted indel frequencies obtained from one of 10 cross-validations (black), which are summarized as one box plot for each model architecture, or to the internal independent test set (red), computed by averaging the predictions obtained from the 10 trained models. The highest two-sided Steiger's test P-value obtained from 10 comparisons, one for each validation set is illustrated on top of the box plots. The boxes represent the first quartile (Q1), the median (internal separator) and the third quartile (Q3); upper whiskers extend up to the last value lower than Q3 +1.5*(Q3-Q1); lower whiskers extend down to the first value greater than Q1+1.5*(Q3-Q1). The suffix 'K' is appended to the model's identifier to designate trees validated with the set of parameters from Kim et al. Two-sided Steiger's test P-values obtained on the

validation sets (from 1 to 10) comparing DeepSpCas9-Val and Sequence-GBRT: 5.77E-03; 1.20E-02; 9.57E-01; 6.87E-04; 6.63E-01; 2.39E-01; 8.73E-03; 5.02E-03; 1.15E-02; 5.46E-02; Sequence-GBRT and Sequence-GBRTK: 3.57E-13; 2.45E-11; 0.00E+00; 2.53E-11; 0.00E+00; 3.78E-10; 3.56E-11; 4.08E-09; 2.66E-15; 1.38E-12; DeepSpCas9-Val and Doench2016-GBRT: 2.50E-04; 8.67E-02; 5.64E-02; 1.64E-02; 8.99E-01; 1.83E-01; 3.63E-03; 3.73E-04; 5.17E-01; 4.70E-03; Doench2016-GBRT and Doench2016-GBRTK: 3.55E-09, 5.86E-11, 2.51E-11, 0.00E+00, 0.00E+00, 8.56E-12, 1.08E-11, 3.09E-08, 1.54E-12, 1.12E-10. P-value computed with the two-sided Steiger's test between DeepSpCas9-Val and Sequence-GBRT: 3.02E-01; DeepSpCas9-Val and Doench2016-GBRT: 1.07E-02. **b.** Generalization performances of DeepSpCas9 and GBRTs, evaluated as Spearman correlation between experimentally measured and predicted indel frequencies. Statistical significance is computed between DeepSpCas9 and GBRTs: two-sided Steiger's test *P < 0.05, NS = not significant. P-values between Sequence-based GBRT and DeepSpCas9 (top to bottom rows): 4.36E-02, 9.18E-01, 8.76E-01, 6.51E-01, 3.21E-01, 6.34E-01; between Doench 2016 GBRT and DeepSpCas9: 5.40E-02, 5.55E-01, 5.85E-01, 5.74E-02, 6.65E-01, 6.23E-01. **c.** DeepSpCas9variants performances decrease after removing non-canonical PAMs. On the X axis PAMs are sorted by median efficiency (left Y axis). Prediction performances (right Y axis) computed for DeepSpCas9variants on the full test set and after removing one PAM at a time, from left to right. The boxes and whiskers are structured as in **a** except for the median, that is here highlighted in red. **d.** Skewed distribution of indel frequencies for gRNAs in the dataset of Wang *et al*. (2019) compared to the Kim *et al*. (2019) and our data.

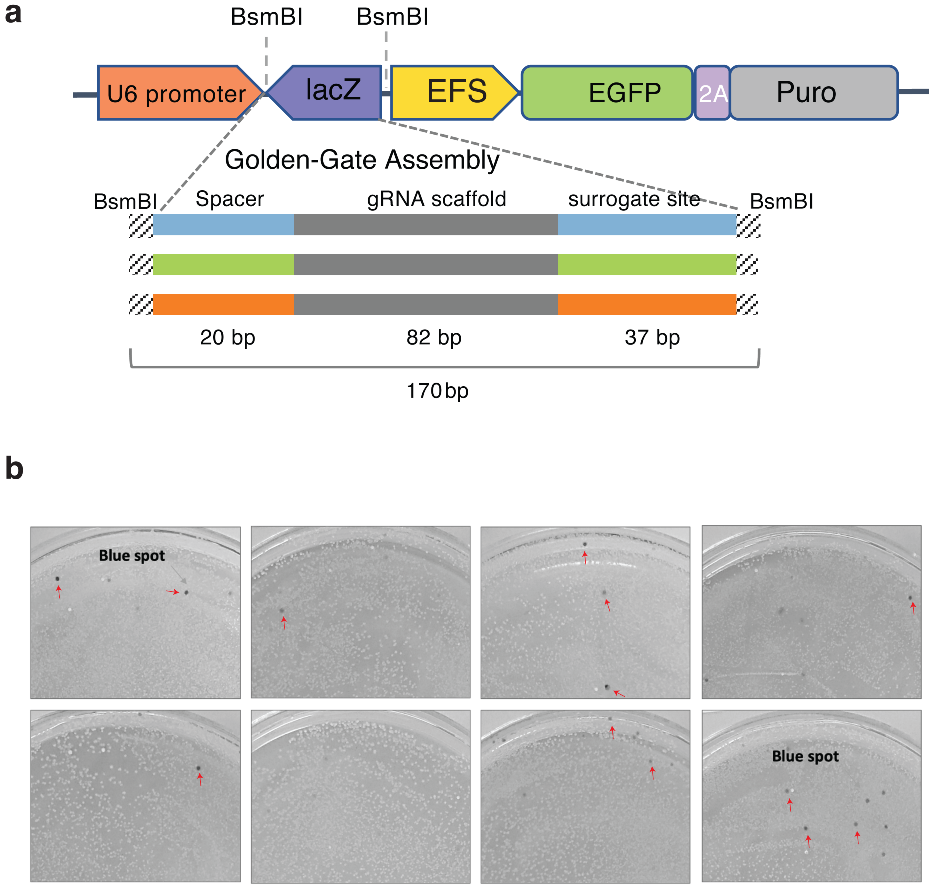

Supplementary Figure 2. Vector design and streamlined vector cloning by Golden Gate assembly. a. Graphical illustration of the lentiviral vector. lacZ, bacterial expression lac operon for rapid and convenient blue-white screen of vector construction efficiency. EFS, EF1alpha promoter (intron-less form, EFS), which controls a polycistronic expression cassette of EGFP and puromycin. **b**. Eight representative plates of transformed *E.coli* cell clones. Ligation product was based on golden-gate assembly of array-synthesized oligos into the empty lentiviral vector (Addgene plasmid # 170459). Representative negative clones are shown with arrows.

a

Endogenous locus

TET2

protospacer PAM

	indel	reads	freq.
TTCAAGAACAGGAGCAGAAGTCACAACAAGCTTCAGTTCTACAGGGATATAAAAATAGAAACCAAGATATGTCTGGTCAAC	indel	reads	freq.
TTCAAGAACAGGAGCAGAAGTCACAACAAGCTTCAGTTCTTACAGGGATATAAAAATAGAAACCAAGATATGTCTGGTCAAC	I1	397777	46.51
TTCAAGAACAGGAGCAGAAGTCACAACAAGCTTCAG--------GGATATAAAAATAGAAACCAAGATATGTCTGGTCAAC	D8	46887	5.48
TTCAAGAACAGGAGCAGGAGTCACAACAAGCTTCAGTTCTTACAGGGATATAAAAATAGAAACCAAGATATGTCTGGTCAAC	I1	37436	4.37
TTCAAGAACAGGAGCAGAAGTCACAACAAGCTTCAGTTC---AGGGATATAAAAATAGAAACCAAGATATGTCTGGTCAAC	D3	21473	2.51
TTCAAGAACAGGGGCAGAAGTCACAACAAGCTTCAGTTCTTACAGGGATATAAAAATAGAAACCAAGATATGTCTGGTCAAC	I1	17925	2.09
TTCAAGAACAGGAGCAGAAGTCACAACA---------------GGGATATAAAAATAGAAACCAAGATATGTCTGGTCAAC	D15	7504	0.87
TTCAAGAACAGGAGCAGAAGTCACAACAAGCTTCAGTTCTTCCAGGGATATAAAAATAGAAACCAAGATATGTCTGGTCAAC	I1	5786	0.67
TTCAAGAACAGGTGCAGAAGTCACAACAAGCTTCAGTTCTTACAGGGATATAAAAATAGAAACCAAGATATGTCTGGTCAAC	I1	4632	0.54
TTCAAGAACAGGGGCAGGAGTCACAACAAGCTTCAGTTCTTACAGGGATATAAAAATAGAAACCAAGATATGTCTGGTCAAC	I1	4455	0.52
TTCAAGAACAGGAGCAGAAGTCACAACAA------------CAGGGATATAAAAATAGAAACCAAGATATGTCTGGTCAAC	D12	3221	0.37

Surrogate locus

TET2

protospacer PAM

	indel	reads	freq.
GCAGAAGTCACAACAAGCTTCAGTTCTACAGGGATAT	indel	reads	freq.
GCAGAAGTCACAACAAGCTTCAGTTCTTACAGGGATAT	I1	558505	53.28
GCAGAAGTCACAACAAGCTTCAG--------GGATAT	D8	57877	5.52
GCAGAAGTCACAACAAGCTTCAGTTC---AGGGATAT	D3	32657	3.09
GCAGAAGTCACAACAAGCTTCAGTTCAAACAGGGATAT	I1	21629	2.06
GCAGAAGTCACAACAAGCTTCA---------------	D15	12177	1.16
GCAGAAGTCACAACA---------------GGGATAT	D15	11370	1.08
GCAGAAGTCACAACAAGCTTCAGTTCTA--------T	D8	5009	0.47
GCAGAAGTCACAACAAG----------------ATAT	D16	4966	0.47
GCAGAAGTCACAACAAGCTTCAGTTCT-----GATAT	D5	4500	0.42
GCAGAAGTCACACCAAGCTTCAGTTCTTACAGGGATAT	I1	4373	0.41

Endogenous locus

CAMK1G

protospacer PAM

	indel	reads	freq.
TCTGCTGCAGCTCCAGAAGTGCTGGCCCAGAAACCCTACAGCAAGGCTGTGGATTGCTGGTCCATCGGCGTCATCACCTAC	indel	reads	freq.
TCTGCTGCAGCTCCAGAAGTGCTGGCCCAGAAACCCTACAAGCAAGGCTGTGGATTGCTGGTCCATCGGCGTCATCACCTAC	I1	239843	46.38
TCTGCTGCAGCTCCAGAAGTGCTGGCCCAGAAACCCTACA---AGGCTGTGGATTGCTGGTCCATCGGCGTCATCACCTAC	D3	15269	2.95
TCTGCTGCAGCTCCAGAAGTGCTGGCCCAGAAACCCTT--GCAAGGCTGTGGATTGCTGGTCCATCGGCGTCATCACCTAC	D2	9108	1.76
TCTGCTGCAGCTCCAGAAGTGCTGGCCCAGAAACCCTACACAGCAAGGCTGTGGATTGCTGGTCCATCGGCGTCATCACCTAC	I2	8831	1.70
TCTGCTGCAGCTCCAGAAGTGCTGGCCCAGAAACCCT-----------GTGGATTGCTGGTCCATCGGCGTCATCACCTAC	D11	6472	1.25
TCTGCTGCAGCTCCAGAAGTGCTGGCCCAGAAACCCTAC--------TGTGGATTGCTGGTCCATCGGCGTCATCACCTAC	D8	3647	0.70
TCTGCTGCAGCTCCAGAAGTGC--------------------AAGGCTGTGGATTGCTGGTCCATCGGCGTCATCACCTAC	D20	3439	0.66
TCTGCTGCAGCTCCAGAAGTGCTGGCCCAGAAACCC------AAGGCTGTGGATTGCTGGTCCATCGGCGTCATCACCTAC	D6	3388	0.65
TCTGCTGCAGCTCCAGAAGTGCTGGCCCAGAAACCCTAC--	D42	3216	0.62
TCTGCTGCAGCTCCAGAAGTGCTGGCCCAGAAACCCTA--GCAAGGCTGTGGATTGCTGGTCCATCGGCGTCATCACCTAC	D2	3192	0.61

Surrogate locus

CAMK1G

protospacer PAM

	indel	reads	freq.
CAGAAGTGCTGGCCCAGAAACCCTACAGCAAGGCTGT	indel	reads	freq.
CAGAAGTGCTGGCCCAGAAACCCTACAAGCAAGGCTGT	I1	222904	44.52
CAGAAGTGCTGGC----------------AAGGCTGT	D16	18499	3.69
CAGAAGTGCTGGCCCAGAAACCCTACA---AGGCTGT	D3	11883	2.38
CAGAAGTGCTGGCCCAGAAACCCTACACAGCAAGGCTGT	I2	10265	2.01
CAGAAGTGCTGGC---------------------TGT	D21	10279	2.01
CAGAAGTGCTGGCCCAGAAACCCT-----------GT	D11	8761	1.73
CAGAAGTGCTGGCCCAGAAAC---ACAGCAAGGCTGT	D3	8640	1.73
CAGAAGTGCTGGCCCAGAAAC--------AAGGCTGT	D8	7245	1.44
CAGAAGTGCTGGCCCAGAAACCCTAC--------TGT	D8	6652	1.33
CAGAAGTGCTGGCCCAGAAACCCTA--GCAAGGCTGT	D2	5506	1.10

b

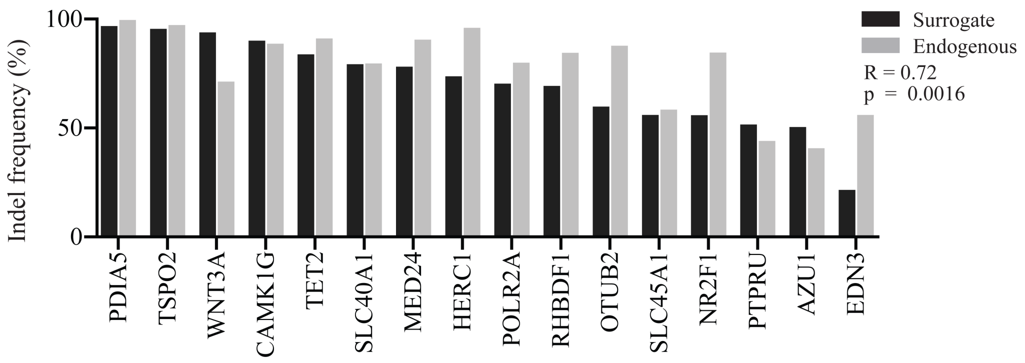

Supplementary Figure 3. Quantification of gRNA editing efficiency in surrogate and endogenous loci. a. Top 10 indel types for surrogate and endogenous locus for two genes (TET2 and CAMK1G). I, insertion. D, deletion. Freq., indel frequency (fraction of total reads). **b**. Total indel frequency for 16 validated loci measured at surrogate and corresponding endogenous locus, Spearman's R.

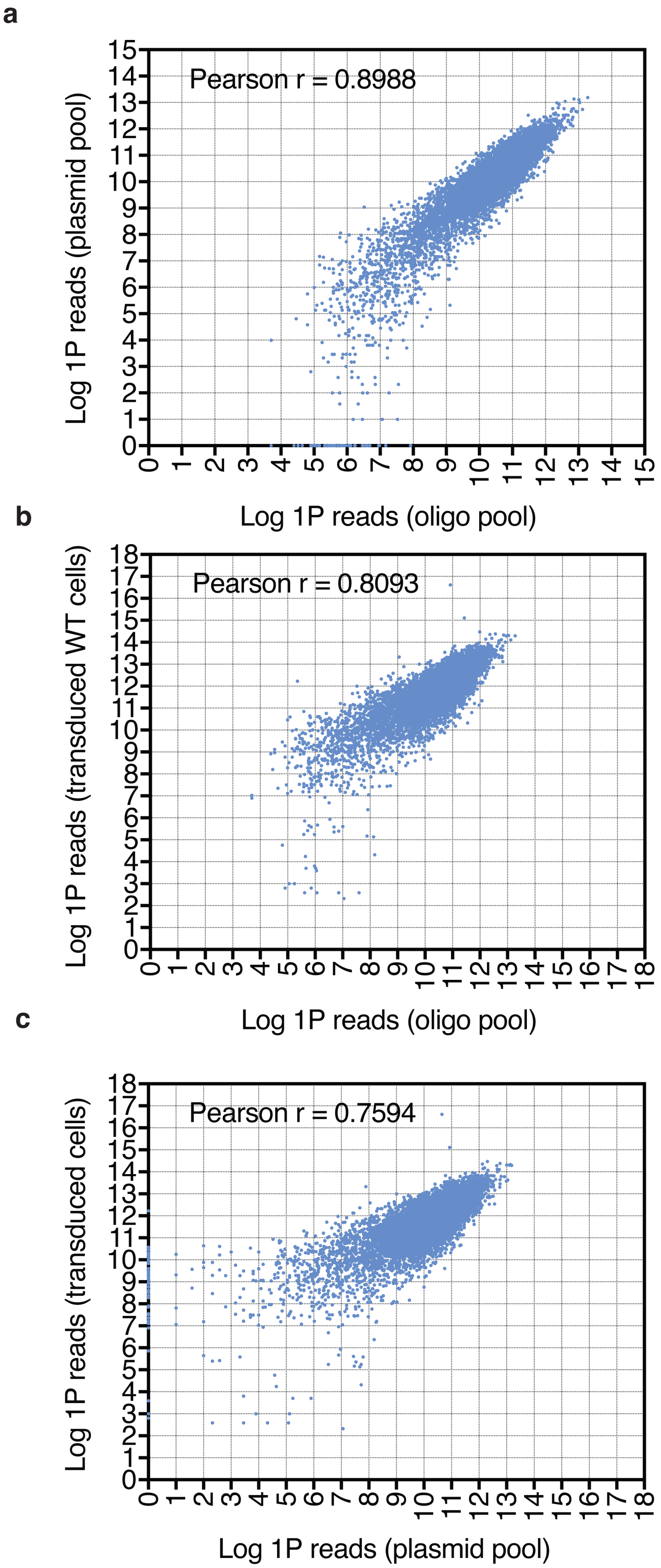
a
Pearson r = 0.8988
Log 1P reads (plasmid pool)
Log 1P reads (oligo pool)
b
Pearson r = 0.8093
Log 1P reads (transduced WT cells)
Log 1P reads (oligo pool)
c
Pearson r = 0.7594
Log 1P reads (transduced cells)
Log 1P reads (plasmid pool)

Supplementary Figure 4. Quality control of surrogate site representation in oligo library, plasmid and lentivirus library by deep sequencing. a. Dot plot of each surrogate site between the oligo pool and the plasmid pool. Log 1P: Log2(total raw reads + 1). **b.** Dot plot of each surrogate site between the oligo pool and lentivirus library transduced WT HEK293T cells (MOI = 0.3, 2 days after transduction). **c.** Dot plot of each surrogate site between the plasmid pool and the lentivirus library transduced WT HEK293T cells (MOI = 0.3, 2 days after transduction). Data for the WT HEK293T cells (MOI = 0.3, 2 days after transduction) are replot as in Supplementary Fig. 6 WT HEK293T cells at day 2. Correlation was provided with Pearson's r.

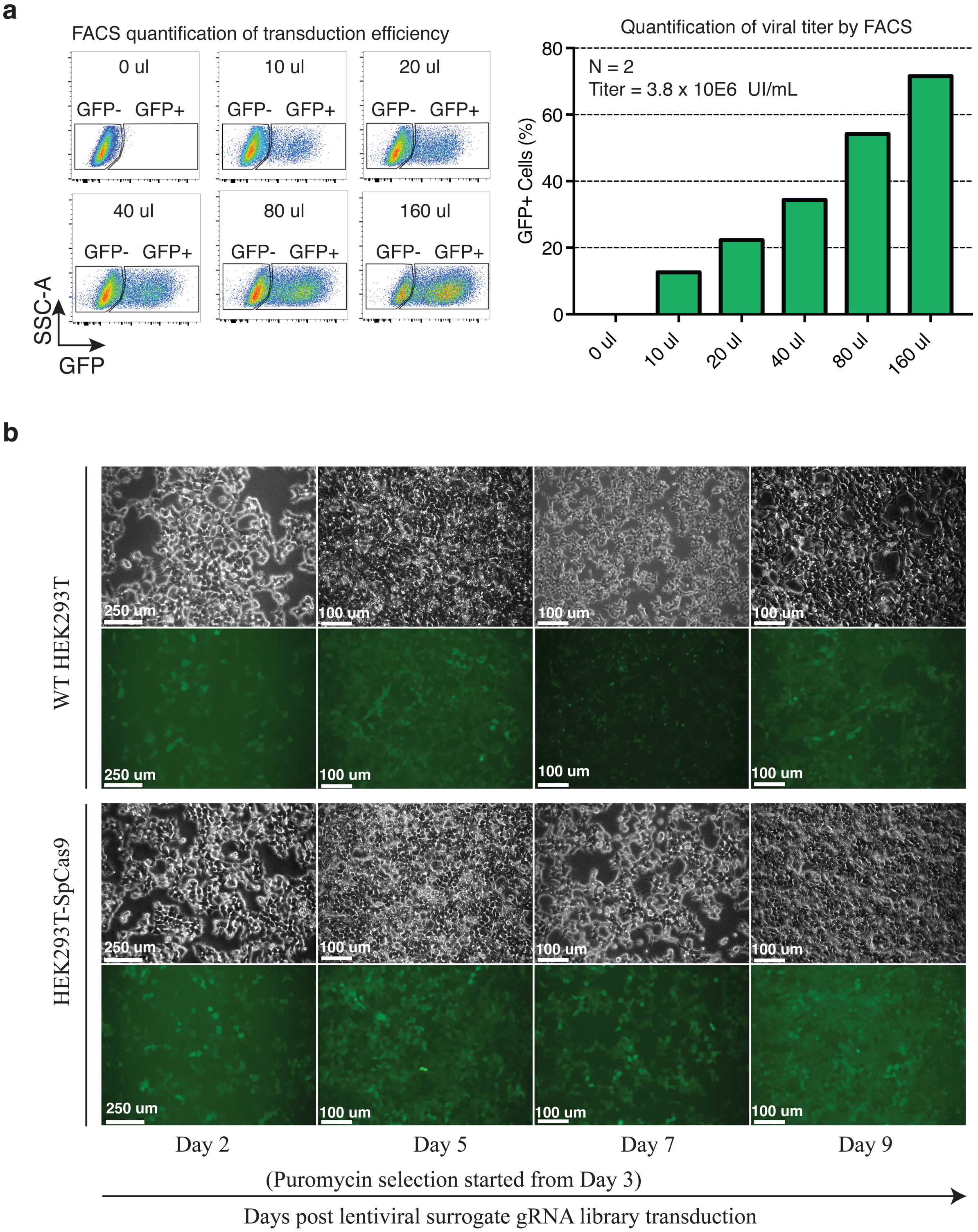

Supplementary Figure 5. Quantification of lentiviral 12K library titer. a. Representative flowcytometry results (n = 2) with gating for GFP positive and GFP negative cells (left) and

quantification of lentiviral functional transduction titer by quantifying mean GFP+ positive cells from duplicates (right). Volumes indicate the amount of crude lentivirus used per transduction. **b.** Representative phase and fluorescence images (n = 3) of HEK293T and HEK293T-SpCas9 cells following lentiviral 12K library transduction (MOI = 0.3) and puromycin selection.

a

b

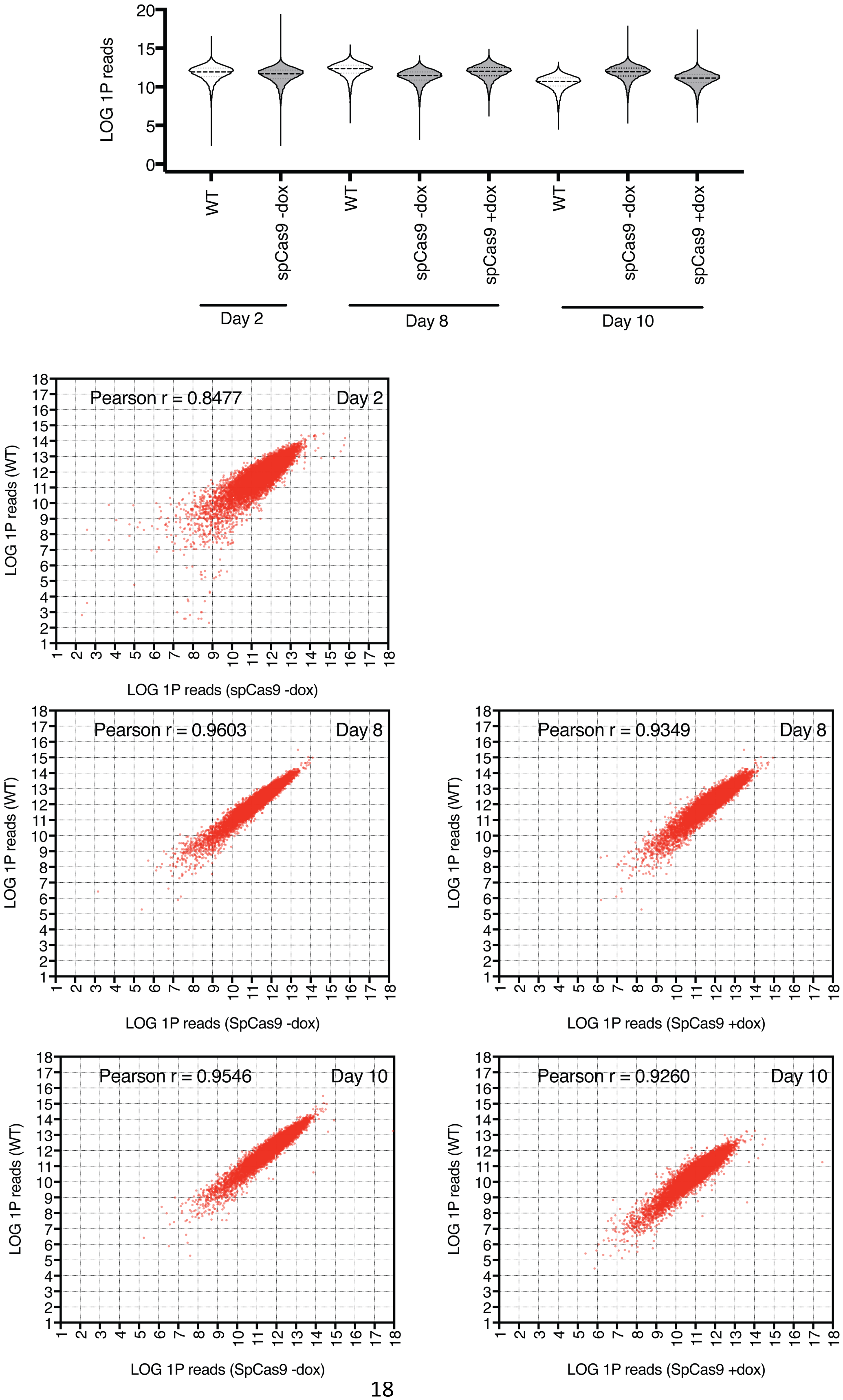
LOG 1P reads
WT
spCas9 -dox
spCas9 +dox
Day 2
Day 8
Day 10
Pearson r = 0.8477
Pearson r = 0.9603
Pearson r = 0.9349
Pearson r = 0.9546
Pearson r = 0.9260
LOG 1P reads (WT)
LOG 1P reads (spCas9 -dox)
LOG 1P reads (SpCas9 -dox)
LOG 1P reads (SpCas9 +dox)

Supplementary Figure 6. Deep sequencing of surrogate loci in lentiviral 12K library transduced WT and SpCas9 cells. a. Violin plot (lines: median and quartiles) of log2 read counts per surrogate site (Log1P, Log2(total raw reads + 1)). **b**. Correlation dot plot of each surrogate site between SpCas9 (- or + dox) and WT cells at 2, 8, and 10 days post transduction.

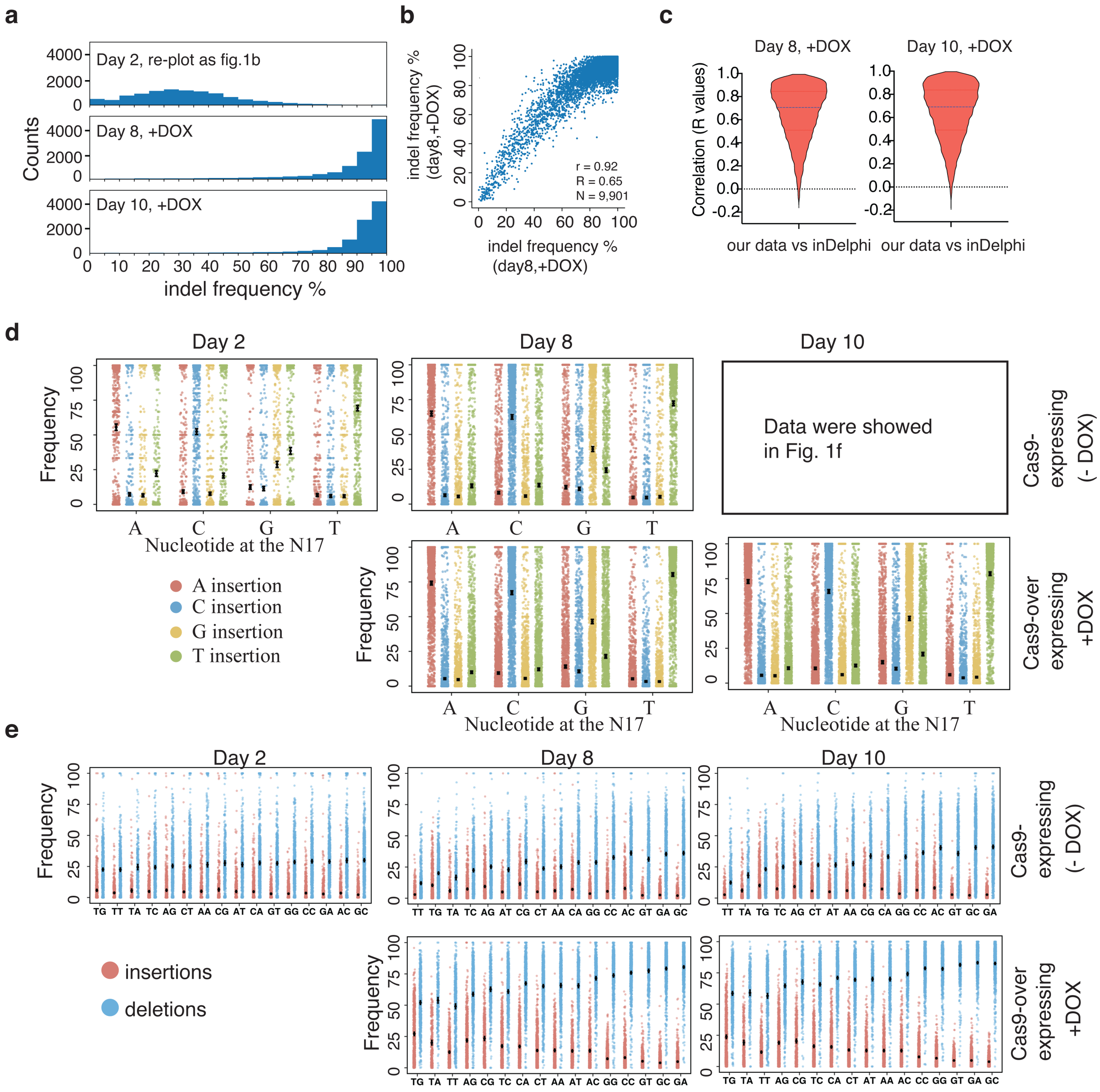

Supplementary Figure 7. Indel profiles captured by targeted deep sequencing. a. Distribution of gRNA efficiency (indel frequency) measured in Day 8-10 cells with Dox addition. **b.** Correlation between indel frequency for gRNAs measured in Day 8-10 cells with Dox addition. **c.** Violin plots (lines: median and quartiles) of Pearson correlation R values between indel profiles captured by targeted deep sequencing of surrogate sites and indel profiles predicted by inDelphi. Each dot represents R value for one surrogate site. N indicates total number of surrogate sites. **d.** Dot plot of 1-bp insertion frequency (error bars are presented with mean ± 95% confidence

interval) among all surrogate sites analyze stratified by N17 nucleotides, N indicates the total number of loci included in the plot. **e.** Dot plot of frequency (error bars are presented with mean ± 95% confidence interval) of deletion and insertion indels stratified by the N17N18 di-nucleotide motifs.

Surrogate site #855

protospacer PAM

TAAGGCTTGCATAGTCCAGTAAGGGTTGGACGGAGGA	indel	reads	freq.
TAAGGCTTGCATAGTCCAGTAAGGGTTTGGACGGAGGA	I1	255	22.11
TAAGGCTTGCATAGTCCAGTAAGGG----ACGGAGGA	D4	208	18.03
-AAGGCTTGCATAGTCCAGTAAGGG----ACGGAGGA	D5	65	5.63
TAAGGCTTGCATAGTCCAGTAAGGGT-GGACGGAGGA	D1	38	3.29
TTTAAGGCTTGCATAGTCCAGTAAGGGTTTGGACGGAGGA	I2	31	2.68
TAAGGCTTGCATAGTCCAGTAAGGGTTGGACG-AGGA	D1	25	2.16
TAAGGCTTGCATAG---------------------GA	D21	24	2.08
TAAGGCTTGCATAGTCCAGTAAGGG--GGACGGAGGA	D2	19	1.64
-AAGGCTTGCATAGTCCAGTAA-------ACGGAGGA	D8	17	1.47
TAAGGCTTGCATAGTCCAGTAA-----------CGGA	D11	16	1.38

Surrogate site #2013

protospacer PAM

TGTCCATCGCCAGCAGCCAGGTGGAGCTGGTGGAAGC	indel	reads	freq.
TGTCCATCGCCAGCAGCCAGGTGGA---------AGC	D9	130	15.58
TGTCCATCGCCAGCAGCCAGGTGGAGCCTGGTGGAAGC	I1	110	13.18
TGTCCATCGCCAGCAGCCAGGTGGAGC----------	D10	62	7.43
TGTCCCATCGCCAGCAGCCAGGTGGAGCCTGGTGGAAGC	I2	39	4.67
-GTCCATCGCCAGCAGCCAGGTGGAGC----------	D11	29	3.47
-GTCCATCGCCAGCAGCCAGGTGGA---------AGC	D10	23	2.75
TGTCCATCGCCAGCAGCCAGGTGGAG---GTGGAAGC	D3	20	2.39
TGTCCCATCGCCAGCAGCCAGGTGG------TGGAAGC	D6/I1	19	2.27
TGTCCATCGCCAGCAGCCAGGTGGAGC-----GAAGC	D5	18	2.15
TGTCCATCGCCAGCAGCCAGGTGGAGC-GGTGGAAGC	D1	16	1.91

Surrogate site #2018

protospacer PAM

AATAGGTAAGGAGACCAGGGGACACAGGCAGGGATGG	indel	reads	freq.
AATAGGTAAGGAGACCAGGGGACACAGGGCAGGGATGG	I1	78	9.00
AATAGGTAAGGAGACCAGGGGACACAG-CAGGGATGG	D1	60	6.92
AATAGGTAAGGAGACCAGGGGACACAGAGGCAGGGATGG	I2	58	6.69
AATAGGTAAGGAGACCAGGG-------------ATGG	D13	51	5.88
AATAGGTAAGGAGACCAGGGGACACAG----------	D10	45	5.19
AATAGGTAAGGAGACCAGGGGA------------TGG	D12	35	4.04
AATAGGTAAGGAGACCAGGGGACACATCCCTGCAGGGATGG	I5/D1	32	3.69
AATAGGTAAGGAGACCA-----------------TGG	D17	27	3.11
AATAGGTAAGGAGACCAGG---------CAGGGATGG	D9	25	2.88
AATAGGTAAGGAGACCAGGGG-------CAGGGATGG	D7	24	2.77

Supplementary Figure 8. Deep sequencing analysis of indel profiles introduced to surrogate sites. Three representative examples of top 10 indels types, reads, and indel frequency measure in HEK293T-SpCas9 cells at day 10. I, insertion. D, deletion. Freq., frequency (fraction of total reads).

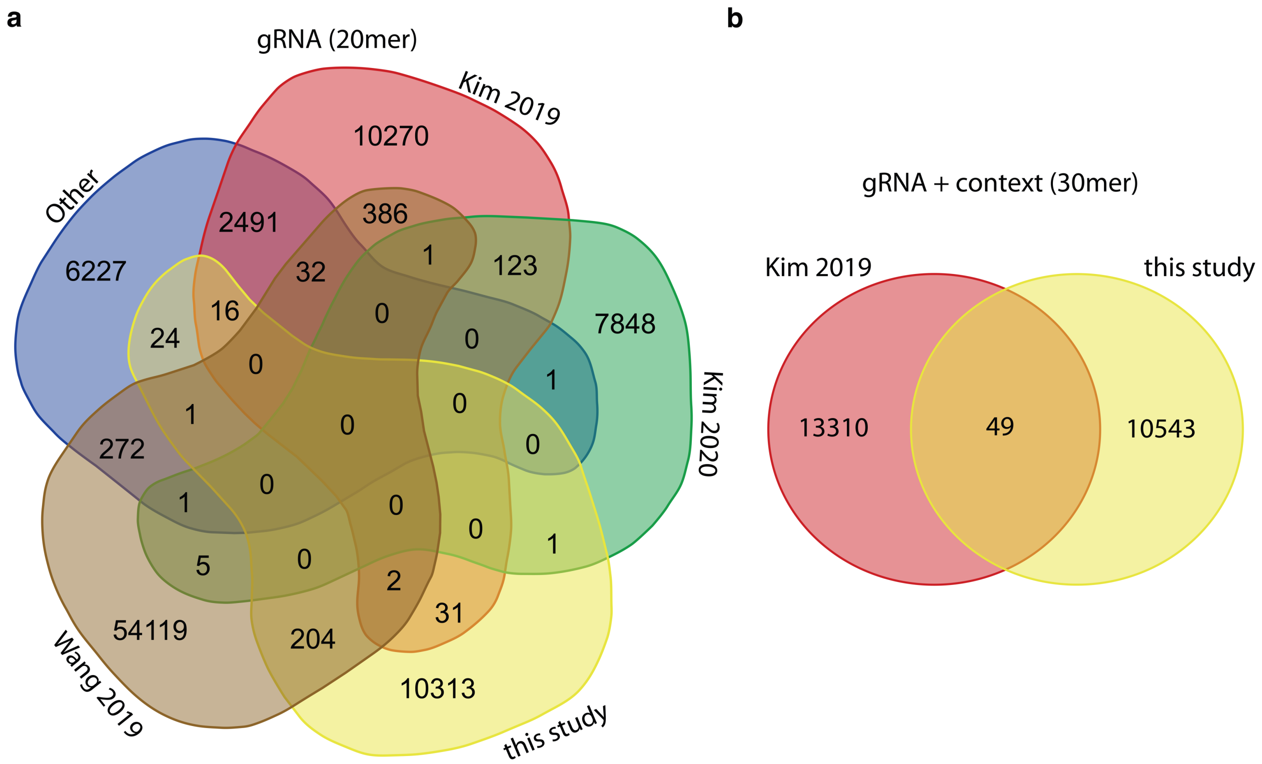

Supplementary Figure 9. gRNAs with measured on-target efficiency. **a**. Venn diagram of the gRNAs (20 nt) for which on-target efficiencies were reported in the datasets included in this study (after data cleaning, see Methods). While the 4 major datasets are shown separately, for visibility the datasets from Chari *et al*., Doench *et al*, Hart *et al*. and Xu *et al*. were grouped together as "Other". **b**. Venn diagram of the gRNA + context (30 nt) sequences for which on-target efficiencies were reported in the datasets of Kim *et al*. (2019) and this study. The union of these two datasets (N=23,902 gRNA + context sequences) was used for the training of CRISPRon. Figure generated using http://bioinformatics.psb.ugent.be/webtools/Venn/.

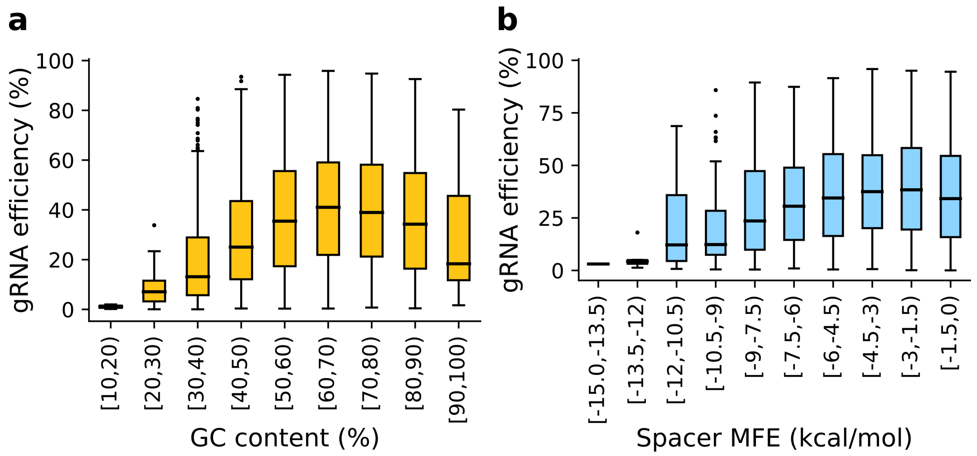

Supplementary Figure 10. Relation between key features of gRNAs and efficiency: **a-b.** Box-plot distribution of gRNAs efficiencies (indel frequencies in cells averaged between day 8 and day 10), split by GC content intervals of 10% (**a**) or by gRNA minimum free energy (MFE) intervals of 1.5 kcal/mol (**b**). In both figures, the boxes represent the first quartile (Q1), the median (thicker line) and the third quartile (Q3); upper whiskers extend up to the last value lower than Q3 +1.5*(Q3-Q1); lower whiskers extend down to the first value greater than Q1+1.5*(Q3-Q1).

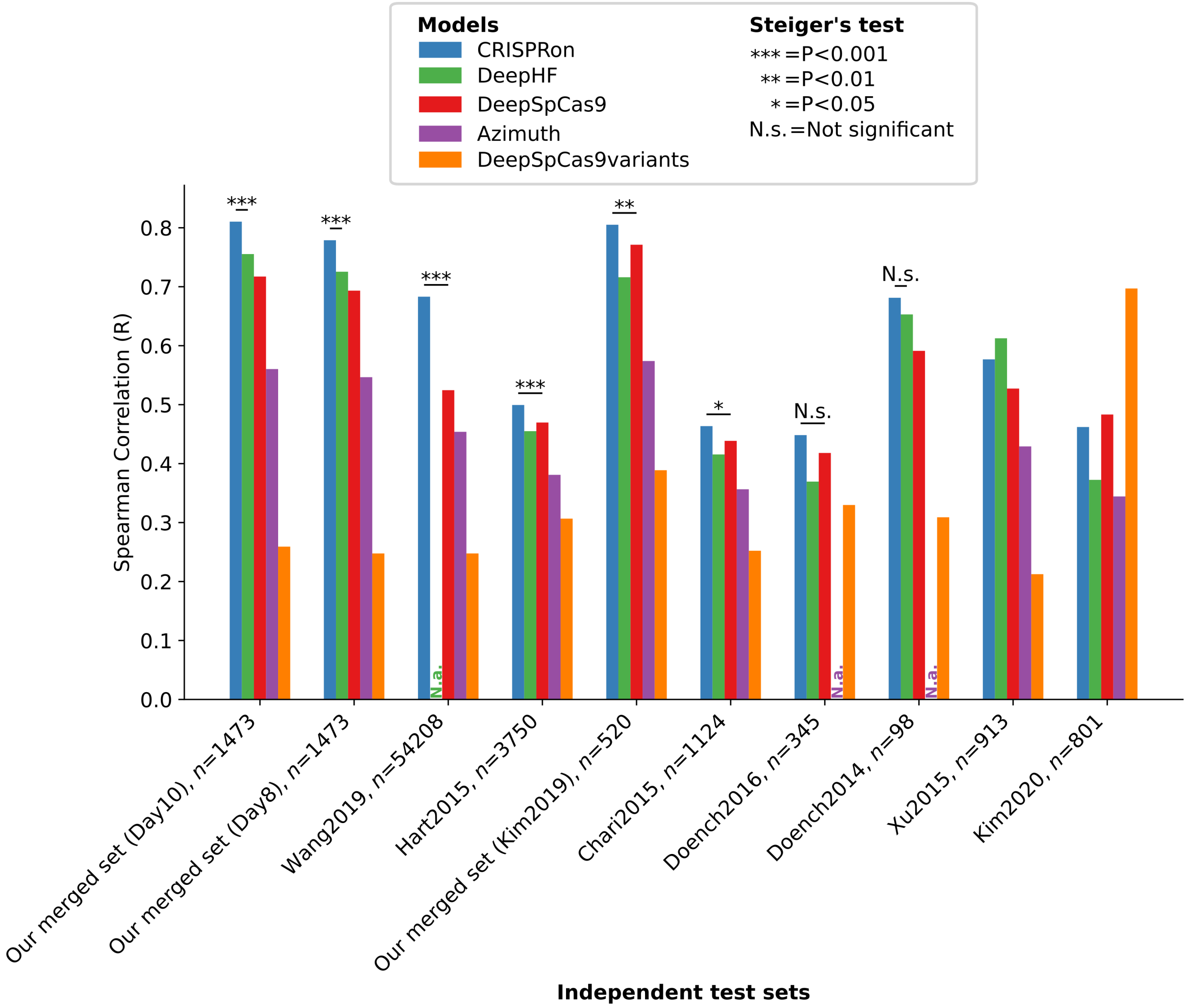

Supplementary Figure 11. Generalization ability of CRISPRon on all independent test sets. Performance comparison between CRISPRon and other existing models on independent test sets. Test gRNAs highly similar to a gRNA in the training sets of any of the models compared (<3 nt difference on 20 nt gRNA) were removed. "N.a."= not available (all gRNAs was regarded as training data due to lack of explicit train-test separation). CRISPRon_v0 was employed for testing on the internal independent test set "Our merged set", for which in this plot Kim *et al.* (2019) and our data were used prior averaging data from multiple days, rescaling, and merging. Our data is split by day (Day 8 and Day10). CRISPRon_v1, or simply CRISPRon, was used for the external independent test sets (for a description of the CRISPRon versions, see Supplementary Table 1). Note that Kim *et al.* (2020) is the internal independent test set of DeepSpCas9variants. Two-sided Steiger's test P-values of all comparisons are reported in Supplementary Data 2.

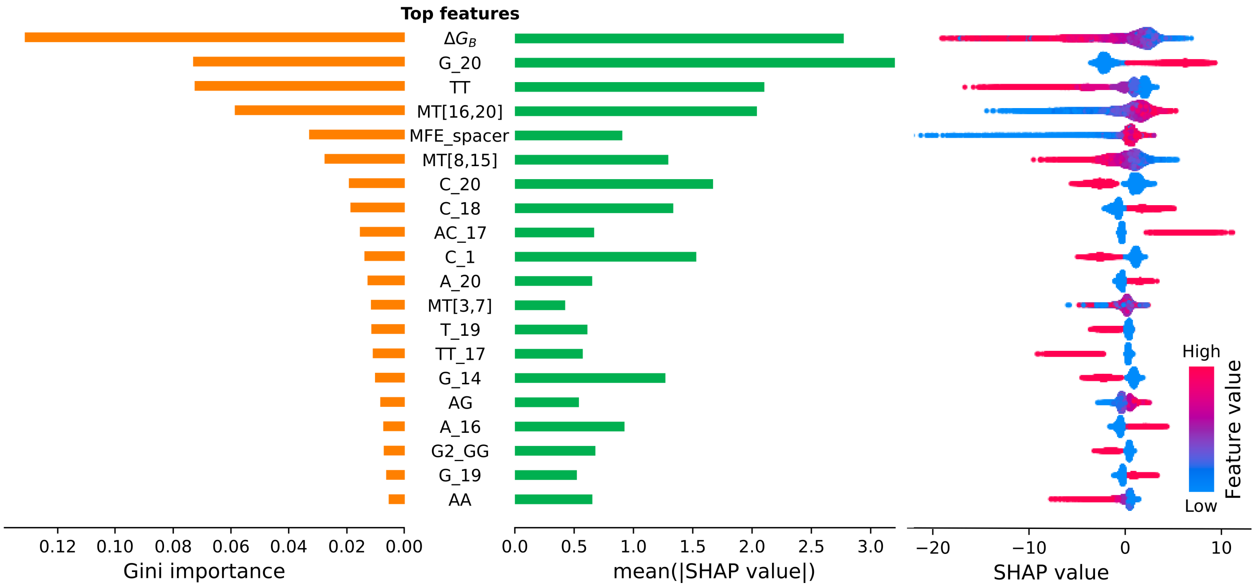

Supplementary Figure 12: Important features associated with gRNA on-target activity. Sequence and thermodynamic features employed to train the CRISPRon-GBRT_v1 were evaluated in terms of Gini (left) and SHapley Additive exPlanations "SHAP" (right) importance. The top 20 common features (among the top 25) identified by the two methods for the GBRT with highest validation performances are displayed. Positions on the 30mer DNA input are labeled as follows: left context: -4 to -1; gRNA spacer: 0 to 20, PAM: N, G1, G2; right context: +1 to +4. The spacer interval used to compute melting temperatures (MT) is indicated in square brackets.

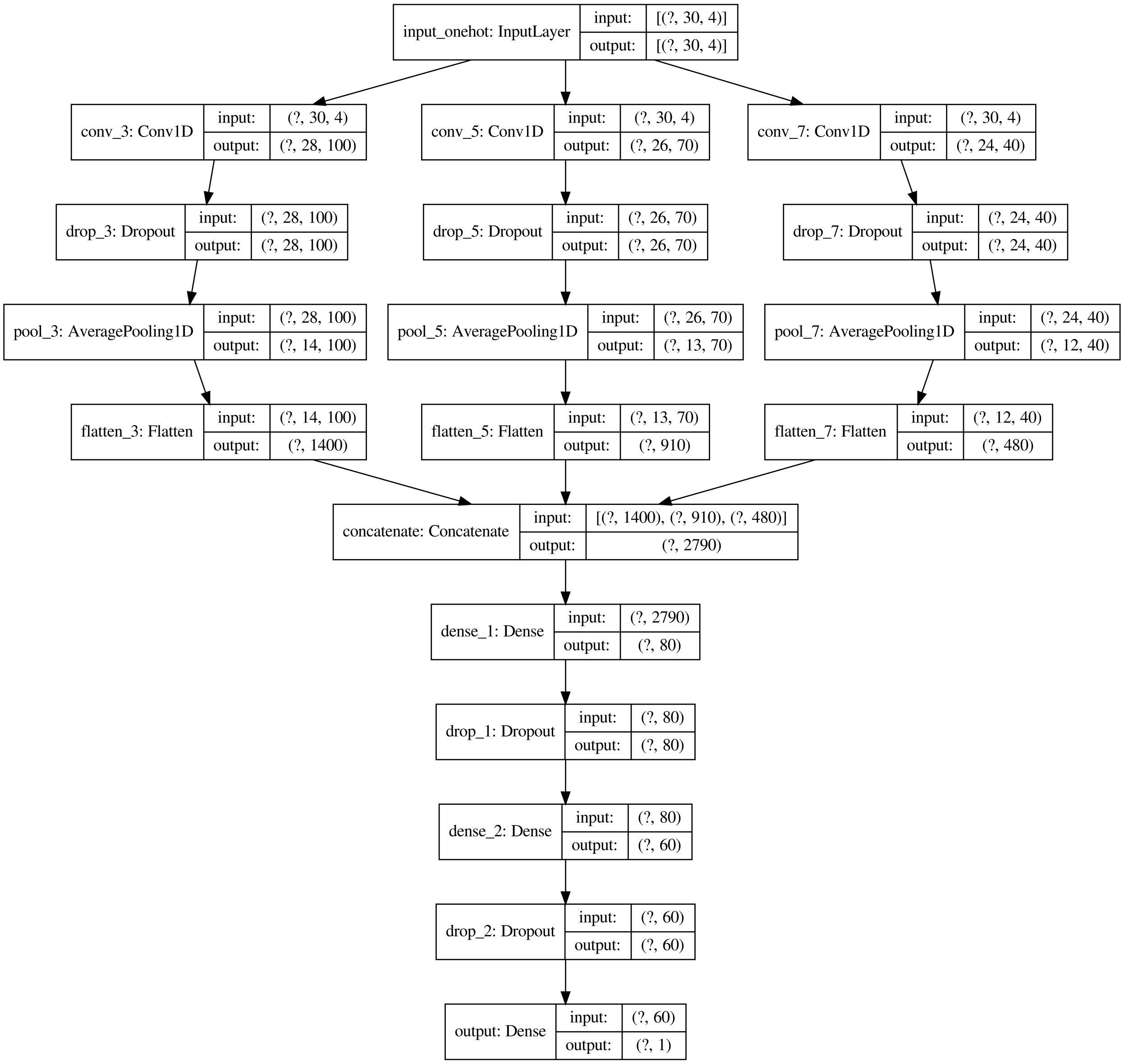

Supplementary Figure 13. Model flow for the deep learning model (C) including convolutions and two dense layers. Technical output obtained from TensorFlow. The input (?, 30, 4) is the one-hot encoded 30mer sequence, where the ? represent the variable number of input sequences in training or evaluation.

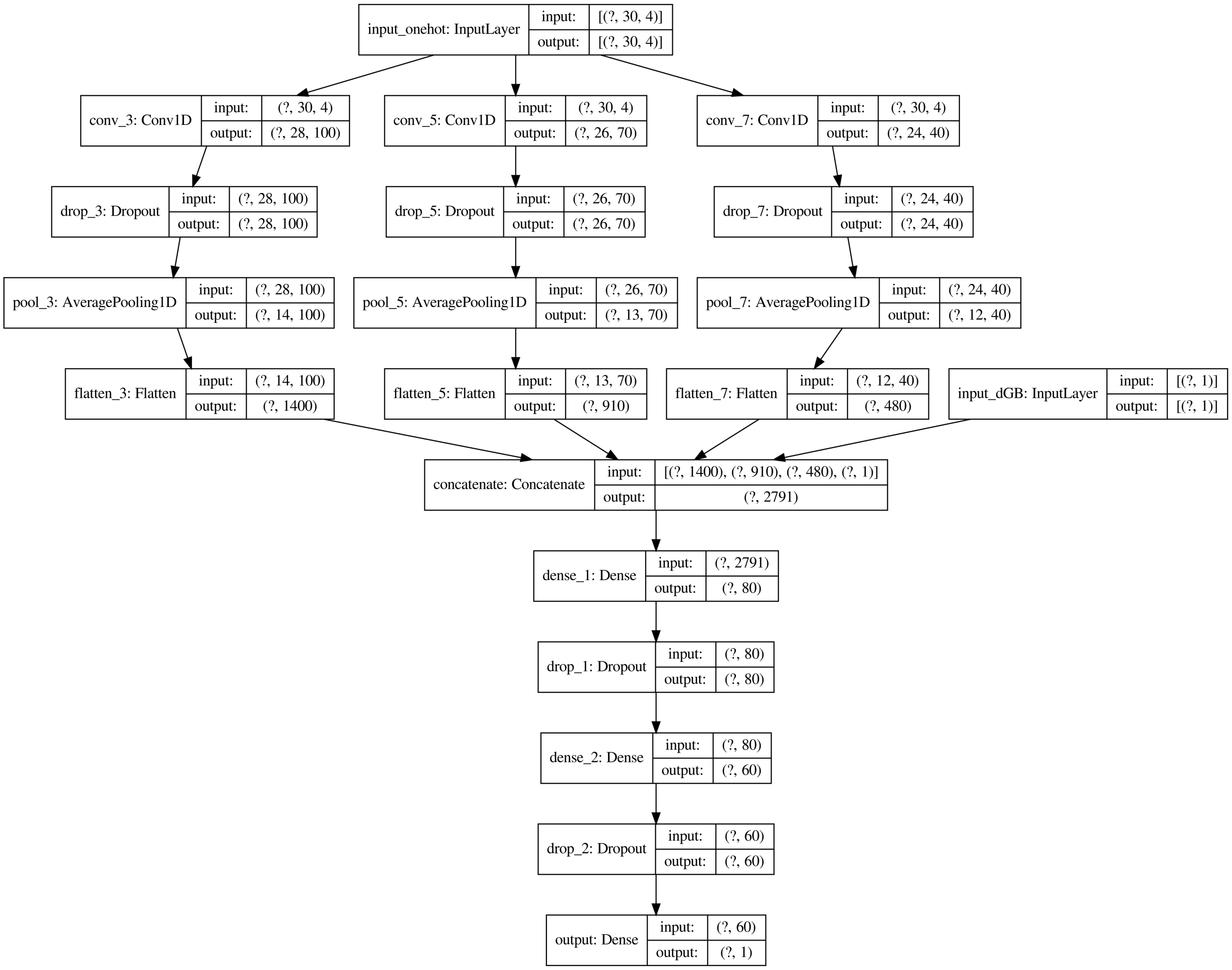

Supplementary Figure 14 Model flow for the deep learning model (CG) including convolutions, two dense layers and ΔG_B. Technical output obtained from TensorFlow. The input_onehot (?, 30, 4) and input_dGB (?, 1) are the one-hot encoded 30mer sequence and the raw unscaled value of ΔG_B, respectively, where the ? represent the variable number of input sequences in training or evaluation.

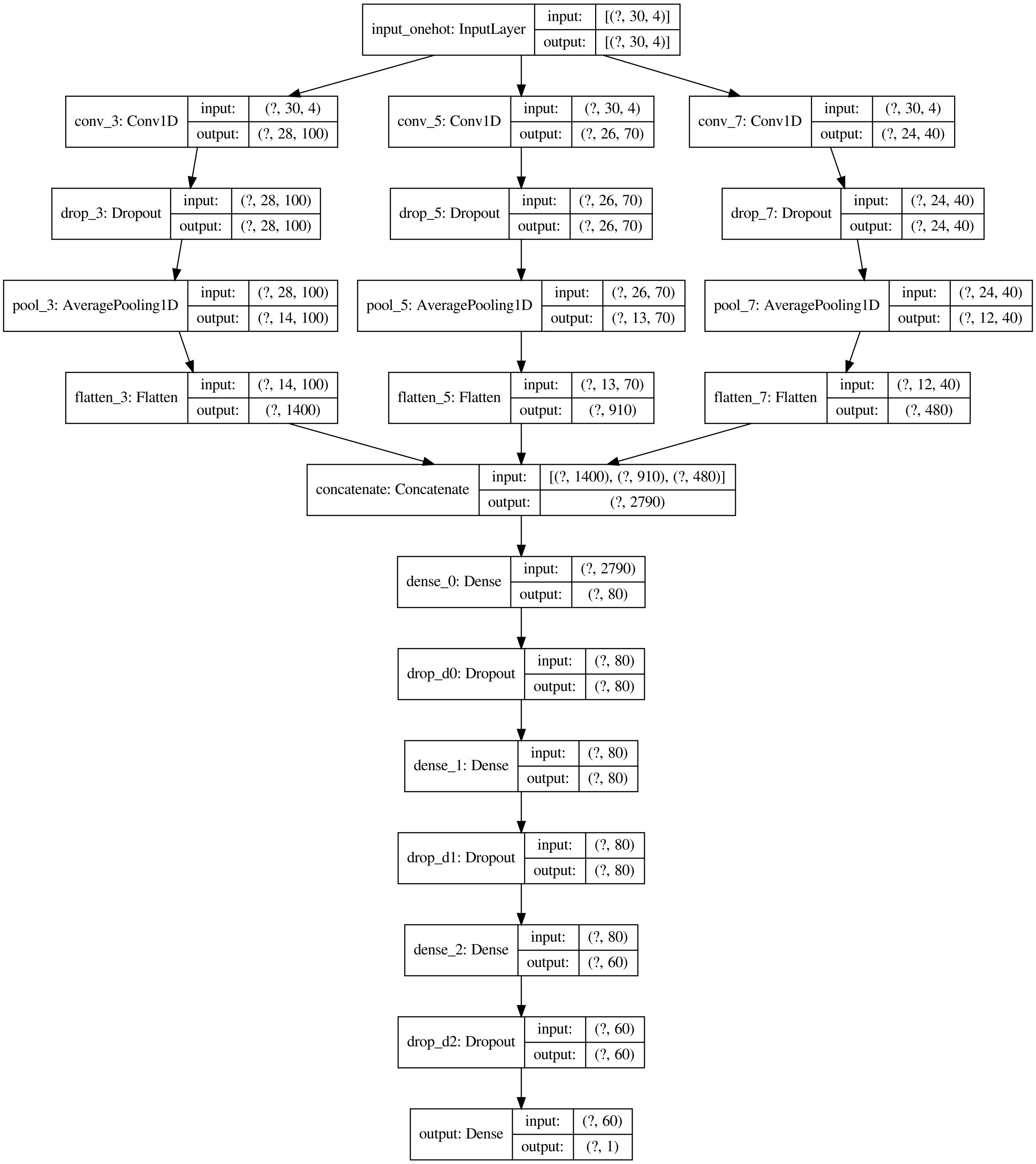

Supplementary Figure 15. Model flow for the deep learning model (Cx) with convolutions and three dense layers. Technical output obtained from TensorFlow. The input_onehot (?, 30, 4) is the one-hot encoded 30mer sequence, where the ? represent the variable number of input sequences in training or evaluation.

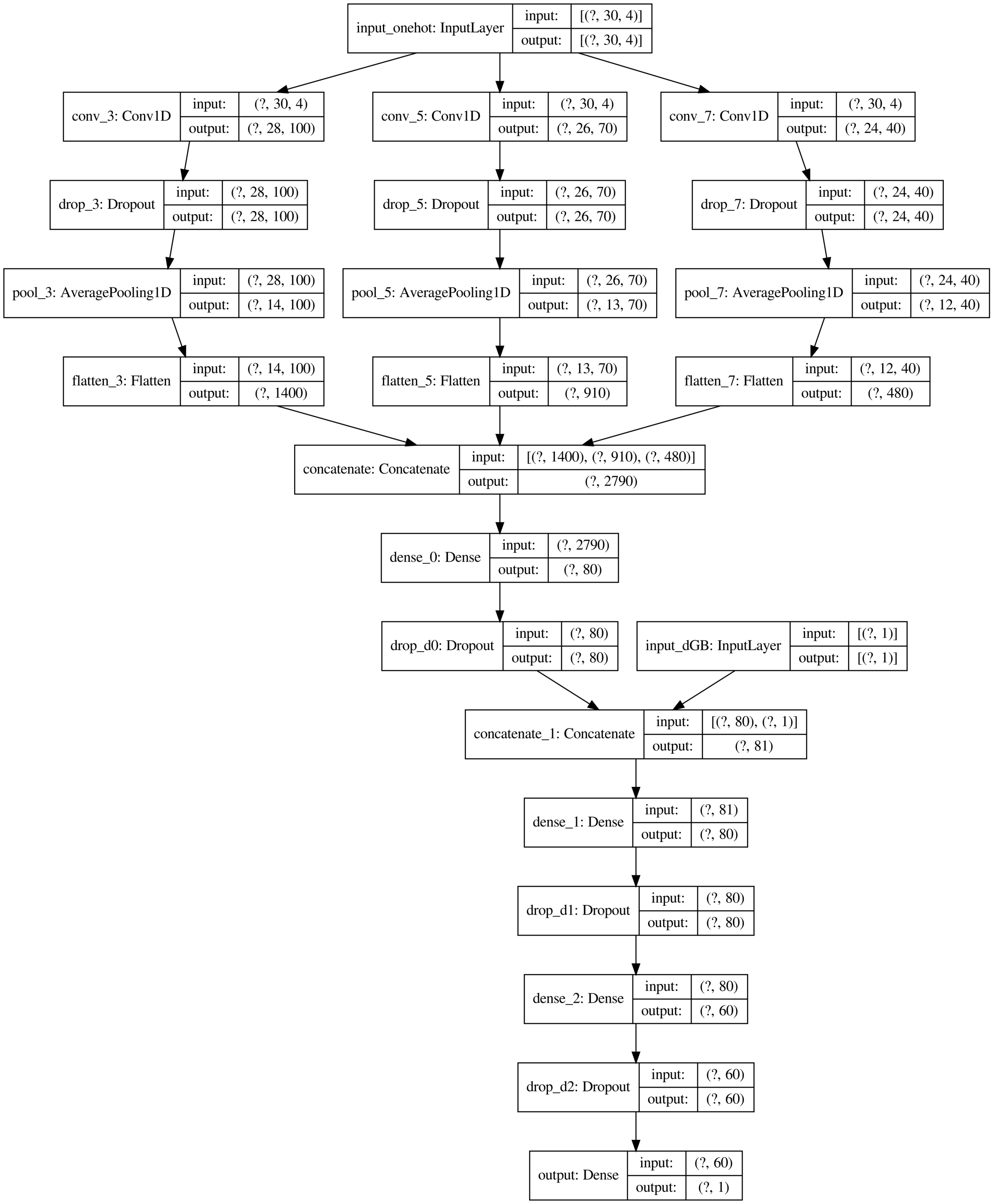

Supplementary Figure 16. CGx model flow including convolutions, ΔG_B and 3 dense layers.

Technical output obtained from TensorFlow. The input_onehot (?, 30, 4) and input_dGB (?, 1) are

the one-hot encoded 30mer sequence and the raw unscaled value of ΔG_B, respectively, where the ? represent the variable number of input sequences in training or evaluation.

Supplementary Data 1. Sequences of 12K surrogate oligonucleotide library, SpCas9 gRNA efficiencies and indel profiles. 12K microarray oligo sequences library (sheet 1), gRNA efficiencies and indel outcomes of gRNAs measured by targeted sequencing at Day 2, Day 8 (Dox+/Dox-) and Day 10 (Dox+/Dox-). **Please see separate table file.**

Supplementary Data 2. Comparison of generalization performances between prediction models. The prediction performances of various models, both from this study and from external ones, are evaluated in terms of Spearman correlation between predicted and actual values. When two or more models are compared, the best result is in bold and the two-sided Steiger's p-value related to the comparison is reported. Underlined values highlight comparisons in which a model from this study showed a statistically significant improvement compared to all other models in the comparison (Steiger's $P < 0.05$). **Please see separate table file.**

Supplementary Table 1. List of machine learning models based on our data. For each model, details about the dataset and the number of its partitions used for model development are given.

**Model name and version**	**Model type**	**Dataset used for model development**	**Num. of dataset's partitions used for model development**
pre-CRISPRon_v0	Deep learning regressor	Our data	5/6
pre-CRISPRon_v1	Deep learning regressor	Our data	6/6
CRISPRon_v0	Deep learning regressor	Our data + Kim *et al.* (2019)	5/6
CRISPRon_v1 (or CRISPRon)	Deep learning regressor	Our data + Kim *et al.* (2019)	6/6
CRISPRon-GBRT_v0	Gradient boosting regressor tree	Our data + Kim *et al.* (2019)	5/6
CRISPRon-GBRT_v1	Gradient boosting regressor tree	Our data + Kim *et al.* (2019)	6/6

Supplementary Table 2. Filters applied to CRISPR gRNA on-target efficiency datasets. The amount of CRISPR gRNAs (30mer) in the on-target efficiency datasets included in this study is reported before and after each applied filter.

	**Doench 2014**	**Doench 2016**	**Chari 2015**	**Hart 2015**	**Xu 2015**	**Wang 2019**	**Kim 2019**	**Kim 2020**	**Our data**
**Initial size**	882	2549	1234	4239	2076	55604	13374	29448 (without gRNAs from tRNA)	D10: 11617<br>D8: 11603<br>Intersection: 11595
**Min200 reads**	-	-	-	-	-	-	-	-	D10: 10933<br>D8: 10655<br>Intersection: 10592
**Not present in hg38**	882	2549	1233	4238	2075	-	-	-	-
**Multimappers hg38**	833	2391	1228	4224	1285	-	-	-	-
**Not overlapping target CDS**	833	2391	1224	4176	1276	-	-	-	-
**Outliers in replicates**	796	2376	-	-	1252	-	-	-	-
**<10 gRNA per target gene**	796	2376	-	-	977	-	-	-	-
**No context defined**	-	-	-	-	-	55022	-	-	-
**Duplicates**	-	-	-	-	-	-	13359	29148	-

**PAM not NGG**	-	-	-	-	-	-	-	8742	-
**Target last 10% CDS**	781	2145	-	4001	971	-	-	-	-
**TOTAL**	**781**	**2145**	**1224**	**4001**	**971**	**55022**	**13359**	**8742**	**10592**

Supplementary Table 3. Hyperparameters selected by GBRT model validation. The hyperparameters used by the GBRTs with the highest Spearman correlation between predicted and actual values, averaged across validation folds, are reported for each model presented in this study. Non-validated default parameters are indicated with "(d)".

**Trained model**	**Hyperparameters**					
	**Learning rate**	**Num. trees**	**Max. depth**	**Min. split**	**Min. leaf**	**Max. features**
CRISPRon-GBRT_v0	0.08	1000	5	20	20	All (d)
CRISPRon-GBRT_v1	0.1	800	5	10	20	All (d)
Sequence-GBRT	0.05	800	5	10	3	All (d)
Doench2016-GBRT	0.1	800	3	10	5	All (d)
Doench2016-GBRT	0.10 (d)	400	600	4	2	Log2
Sequence-GBRTK	0.10 (d)	100	600	2	2	Log2

Supplementary Table 4. Deep learning architectures. Details about each of the deep learning architectures. The models use one input (C, Cx) or two (CG, CGx) inputs. The 30mer sequence is always used as the input for the convolutions of size 3, 5 and 7 of which there are 100, 70 and 40, respectively. The second input is ΔG_B, which is input alongside the output of the convolutions (CG) or alternatively (CGx) the outputs of the convolutions are first collected in a separate dense fully connected layer before combining the output of this dense layer with ΔG_B.

Name	Type	convolutions	dGB	Dense layers
C	Deep	3:100, 5:70, 7:40		80, 60
CG	Deep	3:100, 5:70, 7:40	x	80, 60
Cx	Deep	3:100, 5:70, 7:40		80, 80, 60
CGx	Deep	3:100, 5:70, 7:40	x	80, 80, 60

Supplementary Table 5: Evaluation of gradient boosting and deep learning on an internal independent test set. The merged dataset including our data and that of Kim *et al.* (2019) was split into 6 partitions, 5 of which were used for 5-fold cross-validation while 1 was preserved as internal independent test set. In each column, we list the mean square error (MSE) on the validation set based on the best performing model after 10 repeated initializations (5 for GBRT) using the other for 4 partitions for training. The performance un the independent test set is the performance of the combined model, which is the average of the 5 models build on the validation sets.

**ID**	**Validation1**	**Validation2**	**Validation3**	**Validation4**	**Validation5**	**Validation Av.**	**Indenpendent6**
GBRT	149.67	161.44	159.00	151.63	158.20	155.99	156.93
C	137.17	150.26	146.37	138.68	143.26	143.15	145.79
CG	136.62	148.31	144.26	136.50	143.13	141.76	142.21
Cx	138.21	151.37	148.88	140.21	145.00	144.73	145.12
**CGx**	**134.31**	**147.67**	**144.96**	**135.95**	**141.26**	**140.83**	**140.35**

Supplementary Table 6: Validation of gradient boosting and deep learning. The merged dataset including our data and that of Kim *et al.* (2019) was split into 6 partitions, all of which were employed for 6-fold cross-validation. In each column, we list the mean square error (MSE) on the validation set based on the best performing model after 10 repeated initializations (5 for GBRT) using the other for 5 partitions for training.

**ID**	**Validation1**	**Validation2**	**Validation3**	**Validation4**	**Validation5**	**Validation6**	**Validation Av.**
GBRT	147.45	158.26	154.12	149.78	155.05	158.65	153.89
C	134.29	147.12	142.83	136.38	140.68	146.71	141.33
CG	134.35	144.86	142.52	135.26	139.69	142.64	139.88
Cx	133.77	148.79	145.80	136.97	140.96	147.66	142.33
**CGx**	**130.53**	**144.31**	**139.67**	**133.15**	**138.35**	**141.45**	**137.91**

Supplementary Table 7. Learning rate optimization of deep learning model. Models are as presented in Supplementary Table 5 and are trained on the combined dataset (our data and Kim *et al.* (2019)) using 5-fold cross validation. Each validation set is trained on 10 times and the best performing model is chosen. Reported below are the average of the 5 MSEs on the validation set of the best model trained using the same given validation set. The optimal learning rate was chosen as a compromise between the optimal learning rates obtained for C and CG of 0.0001 and 0.0005, respectively.

Name	Learning rate	Average MSE
C	0.00001	143.51
C	0.00005	143.44
C	0.0001	**143.15**
C	0.0005	144.98
C	0.001	148.19
CG	0.00001	142.29
CG	0.00005	142.07
CG	0.0001	141.76
CG	0.0005	**141.55**
CG	0.001	141.96

Supplementary References

1. Doench, J.G. et al. Rational design of highly active sgRNAs for CRISPR-Cas9-mediated gene inactivation. *Nat Biotechnol* **32**, 1262-1267 (2014).

2. Moreno-Mateos, M.A. et al. CRISPRscan: designing highly efficient sgRNAs for CRISPR-Cas9 targeting in vivo. *Nat Methods* **12**, 982-988 (2015).

3. Labuhn, M. et al. Refined sgRNA efficacy prediction improves large- and small-scale CRISPR-Cas9 applications. *Nucleic Acids Res* **46**, 1375-1385 (2018).

4. Chari, R., Mali, P., Moosburner, M. & Church, G.M. Unraveling CRISPR-Cas9 genome engineering parameters via a library-on-library approach. *Nat Methods* **12**, 823-826 (2015).

5. Wong, N., Liu, W. & Wang, X. WU-CRISPR: characteristics of functional guide RNAs for the CRISPR/Cas9 system. *Genome Biol* **16**, 218 (2015).

6. Chari, R., Yeo, N.C., Chavez, A. & Church, G.M. sgRNA Scorer 2.0: A Species-Independent Model To Predict CRISPR/Cas9 Activity. *ACS Synth Biol* **6**, 902-904 (2017).

7. Rahman, M.K. & Rahman, M.S. CRISPRpred: A flexible and efficient tool for sgRNAs on-target activity prediction in CRISPR/Cas9 systems. *PLoS One* **12**, e0181943 (2017).

8. Peng, H., Zheng, Y., Blumenstein, M., Tao, D. & Li, J. CRISPR/Cas9 cleavage efficiency regression through boosting algorithms and Markov sequence profiling. *Bioinformatics* **34**, 3069-3077 (2018).

9. Muhammad Rafid, A.H., Toufikuzzaman, M., Rahman, M.S. & Rahman, M.S. CRISPRpred(SEQ): a sequence-based method for sgRNA on target activity prediction using traditional machine learning. *BMC Bioinformatics* **21**, 223 (2020).

10. Doench, J.G. et al. Optimized sgRNA design to maximize activity and minimize off-target effects of CRISPR-Cas9. *Nat Biotechnol* **34**, 184-191 (2016).

11. Chuai, G. et al. DeepCRISPR: optimized CRISPR guide RNA design by deep learning. *Genome Biol* **19**, 80 (2018).

12. Kim, H.K. et al. SpCas9 activity prediction by DeepSpCas9, a deep learning-based model with high generalization performance. *Sci Adv* **5**, eaax9249 (2019).

13. Wang, D. et al. Optimized CRISPR guide RNA design for two high-fidelity Cas9 variants by deep learning. *Nat Commun* **10**, 4284 (2019).

14. Kim, N. et al. Prediction of the sequence-specific cleavage activity of Cas9 variants. *Nature Biotechnology* **38** (2020).

15. Haeussler, M. et al. Evaluation of off-target and on-target scoring algorithms and integration into the guide RNA selection tool CRISPOR. *Genome Biol* **17**, 148 (2016).

16. Hastie, T., Tibshirani, R. & Friedman, J. The Elements of Statistical Learning: Data Mining, Inference, and Prediction, Second Edition. (Springer Science & Business Media, 2009).

17. Wang, T., Wei, J.J., Sabatini, D.M. & Lander, E.S. Genetic Screens in Human Cells Using the CRISPR-Cas9 System. *Science* **343**, 80-84 (2014).

18. Xu, H. et al. Sequence determinants of improved CRISPR sgRNA design. *Genome Res* **25**, 1147-1157 (2015).

19. Hart, T. et al. High-Resolution CRISPR Screens Reveal Fitness Genes and Genotype-Specific Cancer Liabilities. *Cell* **163**, 1515-1526 (2015).

20. Xiang, X. & Luo, Y. High throughput quantification of CRISPR gRNA efficiency based on surrogate lentivirus libraries. dx.doi.org/10.17504/protocols.io.bt9jnr4n. (2021).

21. Shen, M.W. et al. Predictable and precise template-free CRISPR editing of pathogenic variants. *Nature* **563**, 646-651 (2018).

22. Lundberg, S.M. & Lee, S.-I. in Advances in Neural Information Processing Systems 30, Vol. 30. (ed. I.G.a.U.V.L.a.S.B.a.H.W.a.R.F.a.S.V.a.R. Garnett) 4765-4774 (Curran Associates, Inc., 2017).

23. Breiman, L. Random Forests. *Machine Learning* **45**, 5-32 (2001).
