# Supplementary material for: Enhancing CRISPR-Cas9 gRNA efficiency prediction by data integration and deep learning
Source: Nat Commun. 2021 May 28;12:3238. doi: 10.1038/s41467-021-23576-0 (PMC8163799; doi:10.1038/s41467-021-23576-0)
Supplement: Supplementary file 3 — Description of Additional Supplementary Files [file 41467_2021_23576_MOESM3_ESM.pdf]

Description of Additional Supplementary Files

Supplementary Data 1. Sequences of 12K surrogate oligonucleotide library, SpCas9 gRNA efficiencies and indel profiles. 12K microarray oligo sequences library (sheet 1), gRNA efficiencies and indel outcomes of gRNAs measured by targeted sequencing at Day 2, Day 8 (Dox+/Dox-) and Day 10 (Dox+/Dox-).

Supplementary Data 2. Comparison of generalization performances between prediction models. The prediction performances of various models, both from this study and from external ones, are evaluated in terms of Spearman correlation between predicted and actual values. When two or more models are compared, the best result is in bold and the two-sided Steiger's p-value related to the comparison is reported. Underlined values highlight comparisons in which a model from this study showed a statistically significant improvement compared to all other models in the comparison (Steiger's $P < 0.05$).
