# Supplementary material for: Enhancing CRISPR-Cas9 gRNA efficiency prediction by data integration and deep learning
Source: Nat Commun. 2021 May 28;12:3238. doi: 10.1038/s41467-021-23576-0 (PMC8163799; doi:10.1038/s41467-021-23576-0)
Supplement: Supplementary file 6 — Reporting summary [file 41467_2021_23576_MOESM6_ESM.pdf]

nature research

Corresponding author(s): Jan Gorodkin, Yonglun Luo

Last updated by author(s): May 2, 2021

Reporting Summary

Nature Research wishes to improve the reproducibility of the work that we publish. This form provides structure for consistency and transparency in reporting. For further information on Nature Research policies, see our Editorial Policies and the Editorial Policy Checklist.

Statistics

For all statistical analyses, confirm that the following items are present in the figure legend, table legend, main text, or Methods section.

n/a	Confirmed	
☐	☒	The exact sample size (*n*) for each experimental group/condition, given as a discrete number and unit of measurement
☐	☒	A statement on whether measurements were taken from distinct samples or whether the same sample was measured repeatedly
☐	☒	The statistical test(s) used AND whether they are one- or two-sided<br>*Only common tests should be described solely by name; describe more complex techniques in the Methods section.*
☒	☐	A description of all covariates tested
☐	☒	A description of any assumptions or corrections, such as tests of normality and adjustment for multiple comparisons
☐	☒	A full description of the statistical parameters including central tendency (e.g. means) or other basic estimates (e.g. regression coefficient) AND variation (e.g. standard deviation) or associated estimates of uncertainty (e.g. confidence intervals)
☐	☒	For null hypothesis testing, the test statistic (e.g. *F*, *t*, *r*) with confidence intervals, effect sizes, degrees of freedom and *P* value noted<br>*Give P values as exact values whenever suitable.*
☒	☐	For Bayesian analysis, information on the choice of priors and Markov chain Monte Carlo settings
☒	☐	For hierarchical and complex designs, identification of the appropriate level for tests and full reporting of outcomes
☐	☒	Estimates of effect sizes (e.g. Cohen's *d*, Pearson's *r*), indicating how they were calculated

Our web collection on statistics for biologists contains articles on many of the points above.

Software and code

Policy information about availability of computer code

Data collection	no software was used.
Data analysis	FlashFry (v 1.80), Fastqc 0.11.3, fastp 0.19.636, FLASh 1.2.137, BWA-MEM , CRISPRoff 1.1.1, RNAfold 2.2.5, Keras/Tensorflow 2.2.0, Python 3.8.3, [illegible]

For manuscripts utilizing custom algorithms or software that are central to the research but not yet described in published literature, software must be made available to editors and reviewers. We strongly encourage code deposition in a community repository (e.g. GitHub). See the Nature Research guidelines for submitting code & software for further information.

Data

Policy information about availability of data

All manuscripts must include a data availability statement. This statement should provide the following information, where applicable:

- Accession codes, unique identifiers, or web links for publicly available datasets
- A list of figures that have associated raw data
- A description of any restrictions on data availability

[illegible]

the the public repository of the Azimuth project at: https://github.com/MicrosoftResearch/Azimuth.
For gRNA selection the drugable gene database was used (http://dgidb.org)
The lentivirus plasmid is made available through Addgene (plasmid #170459)

Field-specific reporting

Please select the one below that is the best fit for your research. If you are not sure, read the appropriate sections before making your selection.

- [x] Life sciences
- [ ] Behavioural & social sciences
- [ ] Ecological, evolutionary & environmental sciences

For a reference copy of the document with all sections, see nature.com/documents/nr-reporting-summary-flat.pdf

Life sciences study design

All studies must disclose on these points even when the disclosure is negative.

Sample size	No sample size calculation was performed.
Data exclusions	The following data exclusion steps have been applied to the filtering of gRNA activity data. (1) Because the estimation of gRNA efficiency will be less accurate if the sequencing coverage is low, we have removed gRNAs supported by less than 200 reads. (2) As gRNA targeting essential genes could potentially cause bias in gRNA efficiency, we have excluded gRNAs targeting essential genes based on a 2-fold enrichment or depletion between SpCas9-expressing cells and MOCK wildtype cells. (3) As DOX induction leads of over-expression of SpCas9 in the HEK293T-SpCas9 cells, the skewed and saturated gRNA efficiency data from SpCas9-over expressing cells from day 8 and day 10 are not suitable for establishing the on-target gRNA activity prediction model. These data are presented in the supplementary materials but excluded from CRISPRon modeling. These data exclusion steps are not pre-established.
Replication	The gRNA efficiencies measured at day 8 and day 10, positively correlated (Pearson's r = 0.91), were averaged. The library contains nearly 12,000 gRNAs, of which gRNA efficiency measured at each sites could be regarded as a test of the gRNA activity quantification method. Systematic benchmarking was carried to validate models. All experiments were performed in at least duplicates and all attempts are successful.
Randomization	This study is not a case-control cohort experiment in life science, randomization was thus not applied.
Blinding	In this study, the data generation and collection were conducted by the Luo group. The data analysis and establishment of the CRISPRon predictor was carried by the Gorodkin group. In-kind blinding strategy was introduced through the independent analyses.

Reporting for specific materials, systems and methods

We require information from authors about some types of materials, experimental systems and methods used in many studies. Here, indicate whether each material, system or method listed is relevant to your study. If you are not sure if a list item applies to your research, read the appropriate section before selecting a response.

Materials & experimental systems

n/a	Involved in the study	
[x]	[ ]	Antibodies
[ ]	[x]	Eukaryotic cell lines
[x]	[ ]	Palaeontology and archaeology
[x]	[ ]	Animals and other organisms
[x]	[ ]	Human research participants
[x]	[ ]	Clinical data
[x]	[ ]	Dual use research of concern

Methods

n/a	Involved in the study	
[x]	[ ]	ChIP-seq
[ ]	[x]	Flow cytometry
[x]	[ ]	MRI-based neuroimaging

Eukaryotic cell lines

Policy information about cell lines

Cell line source(s)	The HEK293T was purchased from ATCC
Authentication	The cell lines were not authenticated.
Mycoplasma contamination	The cell line was tested negative for mycoplasma contamination
Commonly misidentified lines (See ICLAC register)	No commonly misidentified cell lines were used in the study.
